# Modern Sociology

**Published:** 1899-07-29

**Authors:** 


### Modern Sociology.

### THE LAW'S DELAYS.

By Francis Scougall.

Enough has been done—and I must be allowed mildly to express my opinion, somewhat too much—to satisfy the cry for prison reform loudly enunciated by persons, the great majority of whom are practically completely ignorant of the subject. It is to be hoped that they now repose well content on their victorious laurels, for they certainly have triumphantly succeeded in making our English prisons homes of such refinement, comfort, and care that our criminals are now prepared to expect the most affable treatment. So strongly was this felt lately by one of our magistrates, before whom a man was brought for bad conduct in the workhouse, that he said he should be giving no punishment at all if he sent him to one of Her Majesty's prisons, where he would be tended and cared for as a most precious individual, and therefore he should return him to the workhouse, where he hoped some slight restriction might be placed on the *menu* offered for his diet, and that his tastes in the way of amusing conversation might not be too liberally gratified. It will be seen from these remarks on the newly-fashioned criminal jurisdiction that I am not disposed to quarrel with the regulations which formerly embraced the whole course of prison discipline, before our philanthropists arose as an army in warlike array to fight the battle of crime against punishment. Nevertheless I have always been convinced during many years' acquaintance with the treatment of evil doers, that there is one respect in which the law does definitely deal out to them a crying injustice, and I trust that, by ventilating the subject in these columns, I may assist in bringing its details before those whom it most concerns. By a singular coincidence—just as I was entering on a brief exposition of the matter with that view—I saw for the first time an allusion to it in the pages of a London newspaper, which raises a hope that I have chosen a propitious moment for giving here the result of my own experience respecting it. The injustice of which I complain consists in the fact that persons accused of any misdeed for which they are to be tried at the assizes or sessions are sent to prison, there to await the arrival of their judges, if they are not in a position to obtain the benefit of bail. The great majority are utterly unable to secure their temporary release by this means. They generally have no friends willing to become sureties for them, or who, if even ready to take the risk involved, are unable to afford sufficient evidence of substantial power to pay the sums that would be due from them, in the event of the prisoner using his conditional liberty to arrange his own complete disappearance before he is called upon to take his trial. Thus it happens that a very large proportion of persons awaiting judgment have to spend it in prison, under very much the same conditions as if they were already sentenced. Now, as every person is held by law to be innocent until he is definitely convicted of the crime of which he is accused, it is plain that a most serious injustice is involved in his detention in the prison appointed for criminals. The period of his incarceration is sometimes very long, and when his trial does take place, if the evidence proves that he was wrongly accused and he is cleared by a verdict of "not guilty," he will have suffered a heavy punishment while perfectly innocent of any fault which could justify its infliction. A case in point came under the notice of the present writer. A young woman was sentenced to be tried at the next assizes for a serious fraud, of which she had been accused by persons who bore her no good-will. It happened that the judge of the circuit had held his court only the week before, and more than three months must elapse before he again sat in judgment in that city. The accused was quite unable to obtain any substantial help which would have enabled her to be bailed out, and therefore that whole period was passed in prison by her. She was so completely friendless that there was no one sufficiently interested in her to visit her, or send in food to supplement the prison fare on which alone she lived; thus her lot during the period of waiting only varied from that of the convicted criminals round her by her exemption from labour; that, in her case, as in many others, was scarcely a benefit, as she longed for employment to lighten the tedium and monotony of life in the prison cells. It was evident to all who approached her that she was a perfectly respectable, well-conducted young woman, and she steadily maintained from first to last her absolute innocence of the crime attributed to her. Her conduct was perfectly good during the period of her detention, but the bitter sense of disgrace in having become what is called in vulgar parlance "a gaol bird," and the trying conditions of her existence there affected her health and spirits to a very undesirable extent. At length the time for holding the assizes came round again, and the three months' prisoner took her trial with no one to defend her, since she could not afford the help of a counsel. As the result of a careful trial the malice of her accusers, and her own absolute innocence of the crime attributed to her, were triumphantly proved. She was unanimously acquitted by the jury, and told by the judge that she left the court without a stain on her character. None the less had she passed three most miserable months, and went out with the stigma of the "gaol bird" on her, which would seriously stand in the way of her obtaining employment where the circumstances of her detention were no known. This case seems to prove the evils of the system so very clearly that I need not bring forward any more arguments to prove my position, but I am glad to be able to point to a partial remedy which has recently been suggested. The General Council of the Bar have just issued a report in which they draw attention to the evils of the present circuit system; the long detention of accused persons in gaol while awaiting trial is cited by them as one of the most grievous results of the system they condemn, as it is caused by the irregular intervals between the dates of the assizes and the uneven pressure of business on the different occasions. They point out, also, the waste of judicial time and public money from the trial by Supreme Court judges of trivial and unimportant cases, and from the formalities of separate commissions. Were all minor cases summarily disposed of by city and county magistrates, accused persons would seldom be imprisoned while waiting trial for more than a few days, even if the existing circuit system were not altered, and my contention in their favour would thus be met; but there is no doubt that wider improvements on the existing state of judicial arrangements are much to be desired.

